# Efficacy and safety of dihydroartemisinin–piperaquine for treatment of *Plasmodium falciparum* uncomplicated malaria in adult patients on antiretroviral therapy in Malawi and Mozambique: an open label non-randomized interventional trial

**DOI:** 10.1186/s12936-019-2909-5

**Published:** 2019-08-20

**Authors:** Esperança Sevene, Clifford G. Banda, Mavuto Mukaka, Sonia Maculuve, Salésio Macuacua, Anifa Vala, Mireia Piqueras, Linda Kalilani-Phiri, Jane Mallewa, Dianne J. Terlouw, Saye H. Khoo, David G. Lalloo, Victor Mwapasa

**Affiliations:** 1 Centro de Investigação em Saúde de Manhiça (CISM), Maputo, Mozambique; 2 Eduardo Mondlane University, Maputo, Mozambique; 3 University of Malawi, College of Medicine, Blantyre, Malawi; 4 Malawi Liverpool Wellcome Trust Clinical Research Programme, Blantyre, Malawi; 5 Oxford Centre for Tropical Medicine and Global Health, Oxford, UK; 6 Mahidol-Oxford Tropical Medicine Research Unit, Bangkok, Thailand; 7 Institute for Global Health, Universitat de Barcelona, Barcelona, Spain; 8 Liverpool School of Tropical Medicine, Liverpool, UK; 9 University of Liverpool, Liverpool, UK; 10 Tropical and Infectious Diseases Unit, Royal Liverpool University Hospital, Liverpool, UK

**Keywords:** Human immunodeficiency virus, Antiretroviral drugs, Dihydroartemisinin–piperaquine, Malaria, Drug–drug interactions

## Abstract

**Background:**

HIV-infected individuals on antiretroviral therapy (ART) require treatment with artemisinin-based combination therapy (ACT) when infected with malaria. Dihydroartemisinin–piperaquine (DPQ) is recommended for treatment of *Plasmodium falciparum* malaria, but its efficacy and safety has not been evaluated in HIV-infected individuals on ART, among whom drug–drug interactions are expected. Day-42 adequate clinical and parasitological response (ACPR) and incidence of adverse events were assessed in HIV-infected individuals on non-nucleoside reverse transcriptase inhibitor-based ART (efavirenz and nevirapine) with uncomplicated *P. falciparum* malaria treated with dihydroartemisinin–piperaquine.

**Methods:**

An open label single arm clinical trial was conducted in Malawi (Blantyre and Chikhwawa districts) and Mozambique (Manhiça district) involving patients aged 15–65 years with uncomplicated *P. falciparum* malaria who were on efavirenz-based or nevirapine-based ART. They received a directly-observed 3-day standard treatment of DPQ and were followed up until day 63 for malaria infection and adverse events. Day-42 PCR-corrected-ACPRs (95% confidence interval [CI]) were calculated for the intention-to-treat (ITT) population.

**Results:**

The study enrolled 160 and 61 patients on efavirenz and nevirapine-based ART, with a baseline geometric mean (95% CI) parasite density of 2681 (1964–3661) and 9819 (6606–14,593) parasites/µL, respectively. The day-42 PCR-corrected ACPR (95% CI) was 99.4% (95.6–99.9%) in the efavirenz group and 100% in the nevirapine group. Serious adverse events occurred in 5.0% (8/160) and 3.3% (2/61) of the participants in the efavirenz and nevirapine group, respectively, but none were definitively attributable to DPQ. Cases of prolonged QT interval (> 60 ms from baseline) occurred in 31.2% (48/154) and 13.3% (8/60) of the patients on the efavirenz and nevirapine ART groups, respectively. These were not clinically significant and resolved spontaneously over time. As this study was not designed to compare the efficacy and safety of DPQ in the two ART groups, no formal statistical comparisons were made between the two ART groups.

**Conclusions:**

DPQ was highly efficacious and safe for the treatment of malaria in HIV-infected patients concurrently taking efavirenz- or nevirapine-based ART, despite known pharmacokinetic interactions between dihydroartemisinin–piperaquine and efavirenz- or nevirapine-based ART regimens.

*Trial registration* Pan African Clinical Trials Registry (PACTR): PACTR201311000659400. Registered on 4 October 2013, https://pactr.samrc.ac.za/Search.aspx

**Electronic supplementary material:**

The online version of this article (10.1186/s12936-019-2909-5) contains supplementary material, which is available to authorized users.

## Background

Malaria and human immunodeficiency virus (HIV) infections co-exist in most parts of sub-Saharan Africa [1]. Antiretroviral treatment (ART) naïve HIV-infected individuals are more susceptible to *Plasmodium falciparum* malaria infection than the HIV-uninfected population [2, 3]. The World Health Organization (WHO) recommends the use of artemisinin-based combination therapy (ACT) to treat *P. falciparum* malaria infections [4]. Dihydroartemisinin–piperaquine (DPQ) is one of the most commonly used WHO-recommended ACT, but there are few data about its efficacy and safety in those taking ART despite the fact that large numbers of HIV-infected patients on ART are likely to be treated with DPQ for malaria.

Most ART regimens in sub-Saharan Africa contain non-nucleoside reverse transcriptase inhibitors, efavirenz and nevirapine. These drugs are metabolized by the cytochrome (CYP) P450 enzymes, particularly CYP3A4 and CYP2B6, which also metabolize artemisinin-derivatives and piperaquine [5–7]. Thus, co-administration of ART and DPQ may result in drug–drug interactions [5]. Indeed, a recent pharmacokinetic study found that in malaria uninfected HIV-infected individuals on efavirenz-based ART, piperaquine concentrations were 43% lower than ART naïve controls [8]. This drug–drug interaction may compromise DPQ’s efficacy. In addition, malaria uninfected HIV-infected individuals on nevirapine-based ART treated with DPQ had higher piperaquine concentrations than ART naïve controls who received DPQ only [8]. This drug–drug interaction may increase piperaquine-related adverse events.

In view of the limited data on the efficacy and safety of DPQ in HIV-malaria co-infected patients, a single arm clinical trial was conducted to estimate the efficacy of DPQ when used to treat parasitologically-confirmed uncomplicated clinical *P. falciparum* malaria in HIV-infected people on standard ART regimens (efavirenz- or nevirapine-based ART). Specifically, the trial assessed the day-42 PCR-corrected adequate clinical and parasitological response [ACPR] to examine whether it exceeds 90%, the WHO recommended benchmark for an efficacious anti-malarial drugs [9]. In addition, the trial assessed the safety of DPQ by determining the incidence of adverse events.

## Methods

### Study sites and study population

This study was part of a multi-country single arm clinical trial aimed at assessing the efficacy and safety of two artemisinin-based combinations (DPQ and artemether–lumefantrine) when used to treat malaria in HIV-infected adults on standard ART. One of the single arm trial assessing the efficacy and safety of AL was conducted in Zambia and findings have been reported elsewhere [10]. In this paper, findings from another single arm trial assessing the efficacy and safety of DPQ which was conducted from October 2013 to June 2015 at Queen Elizabeth Central Hospital and Chikhwawa District Hospital in Malawi as well as Manhiça District Hospital in Mozambique, are reported. These are settings of moderate-high transmission of malaria [11, 12] and high HIV prevalence [13, 14]. Blantyre is an urban district in Southern Malawi with an estimated population 1,239,647, while Chikhwawa is a rural district located 34 km south of Blantyre with an estimated population of 518,284. In 2014, the malaria parasite prevalence in under-five children in Malawi was 33% and higher in rural (37%) than urban areas (11%) [11]. HIV prevalence in Malawi was estimated at 10.6% in 2015 [13]. Manhiça is a rural district in Southern Mozambique located 80 km north of Maputo city, with an estimated population of 178,000 in 2014. Malaria parasite prevalence in under-five children was estimated at 51% in 2013 [12] while HIV prevalence in the district was estimated at 39.9% in 2010 [14].

During the study period, the criteria for initiating ART in the two countries were WHO HIV disease stages 3 or 4, CD4 cell count < 350, pregnancy or lactation [15]. In October–December 2013, > 87% of ART individuals in Malawi were on a fixed dose combination of tenofovir/lamivudine/efavirenz while 6% were on stavudine/lamivudine/nevirapine or zidovudine/lamivudine/nevirapine [16]. In Mozambique, the majority of the ART individuals were on fixed dose zidovudine/lamivudine/nevirapine but the use of fixed dose tenofovir/lamivudine/efavirenz increased steadily over the study period. In both countries, artemether–lumefantrine was the first-line treatment for uncomplicated malaria. DPQ was registered but not routinely available within the public health system in the two countries. Nevertheless, it was evaluated in this study because it is one of the WHO recommended artemisinin-based combinations and has a more convenient dosing schedule than AL (once daily for 3 days). The efficacy and safety of AL was assessed in a separate trial [10].

### Study design and clinical procedures

This was a single arm clinical trial (Registration number: PACTR201311000659400). HIV-infected patients on nevirapine- or efavirenz-based ART suspected of having malaria were pre-screened through history taking and clinical examination to determine their eligibility for the study. The study inclusion criteria were as follows: age ≥ 15 to ≤ 65 years; weight ≥ 35 kg; documented fever (axillary ≥ 37.5 °C) or history of fever 24 h prior enrolment; smear positive *P. falciparum* malaria monoinfection with asexual malaria parasite densities < 200,000/µL; ability to swallow oral medications and willingness and ability to comply with scheduled visits, supervised treatment administration, laboratory tests, and other study procedures. The following were the exclusion criteria: severe malaria as per WHO criteria [17]; mixed infection with another *Plasmodium* species; haemoglobin (Hb) concentration < 7 g/dL; severe sickle cell disease or sickle-haemoglobin C anaemia; evidence of pregnancy or lactation; use of any anti-malarial treatment or drug with anti-malarial activity within the past 1 month, except cotrimoxazole; history of DPQ hypersensitivity reactions; gastrointestinal diseases that could alter gut absorption or motility; history of splenectomy; history of epilepsy or convulsions; pre-existing clinically-significant cardiac, liver, renal, neurological or psychiatric abnormalities; alternative clinical cause of fever other than malaria and participation in any investigational drug study in the past 30 days.

Finger-prick blood samples were taken from those who satisfied the preliminary eligibility criteria and tested for malaria using Rapid Diagnostic Test (RDT) (SD BIOLINE Malaria Ag P.f/Pan test produced by Alere) and for haemoglobin concentration using Hemocue Haemoglobinometer. Thick blood smear microscopy examinations were performed on patients with RDT positive malaria while clinical examinations were performed in those with confirmed malaria parasitaemia. Consenting participants were enrolled and scheduled for a 3-day hospital admission.

The participants received dihydroartemisinin–piperaquine (Eurartesim^®^, Sigma Tau): 3 tablets for study participants < 60 kg or 4 tablets study for participants ≥ 60 kg. Each tablet contained dihydroartemisinin/piperaquine 40 mg/320 mg, respectively, administered at 0 h, 24 (+ 4) and 48 (+ 4) h after the first dose. Participants’ vital signs were measured at 6-hourly intervals and adverse events were monitored. A 12-Lead electrocardiogram (ECG) was performed before the first dose of DPQ and within 2 h after administration of the third dose DPQ. Any patient with Fridericia-corrected QT (QTcF) interval of ≥ 450 ms or QTc increase of > 60 ms from the baseline underwent follow-up ECGs until resolution of the abnormality. Participants were discharged at least 24 h after taking the third (last) dose of DPQ (post-treatment day 3) and advised to come for follow up visits on post-treatment days 7 (± 1), 14 (± 1), 21 (± 2), 28 (± 2), 35 (± 2), 42 (± 2) and 63 (± 2). Participants were encouraged to return to the health facility any time they felt unwell (unscheduled visits). All adverse events were graded using the DAIDS criteria [18]. Adverse events with onset or increased severity after the first dose of DPQ were counted as treatment-emergent adverse events (TEAEs). During follow up visits, participant’s time and any incurred expenses when attending the study clinic were appropriately compensated, as approved by the ethics committees.

### Laboratory procedures

During the admission period, thick blood slides were collected pre-dosing and at 6-hourly intervals until after obtaining two consecutive malaria negative smears. The slides were also collected at scheduled and unscheduled follow-up visits. The slides were Giemsa-stained and read by an experienced microscopist using standard protocols [19]. For quality control, all slides were re-read by a second microscopist; a third microscopist settled any discrepant readings. Dry blood spot (DBS) samples were collected on filter paper (Whatman 3MM^®^) at baseline and during recurrent malaria episodes. Parasite DNA was extracted from the DBS samples, amplified using polymerase chain reaction (PCR) and genotyped for merozoite specific protein (MSP) 1 and 2 to distinguish malaria recrudescence from re-infection, using methods previously described [20]. Samples that did not produce results were classified as indeterminate.

Venous blood samples were collected on days 0, 3, 28, 42 and 63 for biochemistry tests using a Beckman CX5^**®**^ Chemistry analyzer, on days 0, 3, 7, 28, 42 and 63 for haematological tests using a Beckman Coulter^**®**^ HMX Analyzer and on days 0, 28, and 63 for CD4 cell count measurement using a BD FACSCount™ machine. Plasma samples collected on days 0, 28, and 63 were stored for future HIV viral load assays.

Blood samples for sparse pharmacokinetic (PK) assays were collected in sampling windows of 0–6, 6–48, 48–60 h, and on days 7, 21, 28 or 35 from first dose, as previously recommended [21]. In this paper, the relationship between day-7 concentrations and ACPR was explored since day-7 concentrations of the slowly eliminated partner drug of ACT have been shown to be a better determinant of therapeutic response than the area under the concentration–time curve [22]. The PK samples were analysed using a previously described HPLC–UV assay method [8]. The lower limit of quantitation (LLOQ) of the piperaquine was 25 ng/mL, with a coefficient of variation of < 10%. The PK laboratory at the Malawi-Liverpool Wellcome Trust Clinical Research Programme in Blantyre, Malawi, participated in the World Wide Antimalarial Resistance Network’s external quality assurance programme [23].

### Study endpoints

The primary study endpoint was proportion of patients with PCR-corrected day 42 ACPR, defined as patients who did not have parasitaemia on day 42 that exhibited identical *P. falciparum* malaria PCR markers (merozoite surface protein 1 and 2) with those at baseline, irrespective of axillary temperature, and who had not previously met any of the criteria of early treatment failure (ETF), late clinical failure (LCF) or late parasitological failure (LPF). Standard WHO definitions of ETF and LCF were used [9].

The other primary study end points were grade 3 or 4 TEAEs of special interest (Fridericia-corrected QT interval prolongation, dizziness, palpitations, urticaria or itchiness) and serious adverse events (SAEs) as per standard definitions [24]. Local study physicians determined the relationships between DPQ and the adverse events (AEs). A Data Safety and Monitoring Board reviewed serious AEs and adverse events of special interest (AESIs) and assessed the validity of study physicians’ decisions. Secondary endpoints, included day 42 PCR-uncorrected ACPR, time for parasite to decline by 50% (PC_50_) and 90% (PC_90_), fever clearance time and trends in haemoglobin concentrations and CD4 cell counts from baseline to day 28.

### Samples size

Sample size calculation was based on estimates of total treatment failure rate (TTFR). The estimated day-42 PCR corrected TTFR was ≤ 10% [25]. A precision of 5%, around this point estimate, allowed the upper limit of the 95% Wald binomial confidence interval to be 15%. Using the formula for estimating a single study population sample size [26], our effective sample size was estimated at 138 for each ART type. The final sample size, for each ART group, was 163 after adjusting for an anticipated loss-to-follow-up rate of 15%. This sample size was achieved for the efavirenz-ART group but not for nevirapine-ART group, as the ART programs in both countries had successfully transitioned from nevirapine- to efavirenz-based ART first-line regimens. Sample size calculations including all subsequent statistical analysis were performed in STATA 13.1

### Statistical analyses

For the primary end-point, three analysis populations were used. Firstly, the Intention-to-treat (ITT) population included patients who received at least 1 dose of study medication. Secondly, the per-protocol (PP) population included all participants who had the primary endpoint data at day 42, received a full course of DPQ and adhered to the follow-up visit schedule. Thirdly, the safety population included all patients who received any amount of study medication and had at least 1 assessment after dosing. ACPR plus 95% CI in PP and ITT populations were calculated. Sensitivity analyses were performed using the ITT and PP populations that first considered all participants with missing data as having parasitological failure and then considered the same participants as having treatment success.

Statistical analyses for secondary ACPR endpoints were similar to the primary endpoints. In addition, the Kaplan–Meier survival plots were used to summarize the time to PCR-corrected and uncorrected treatment failure. Parameters assessing post-treatment parasite clearance (PC_50_, PC_90_ and parasite clearance half-life) in the two ART groups were estimated using the WorldWide Antimalarial Resistance Network parasite clearance estimator, as described elsewhere [27].

Descriptive statistics were computed for baseline variables in the two ART groups. However, as this study was designed to estimate and not to compare efficacy and safety of DPQ between the two ART groups, no formal statistical comparisons of baseline characteristics efficacy or safety endpoints were made between the two ART groups. As part of a priori exploratory analysis, and where appropriate, Wilcoxon rank-sum/Mann–Whitney U test was used to compare distributions of the day-7 piperaquine concentrations in those who attained or did not attain ACPR by day 42. Piperaquine concentrations below the LLOQ were imputed to half the lower limit of quantification and included in the estimation of median piperaquine exposure if the imputed values were < 10% of the data. Additionally, Wilcoxon matched paired signed-rank test was used to compare baseline and day 28 CD4 cell and haemoglobin values in each ART group.

## Results

### Study profile

**Fig. 1:**
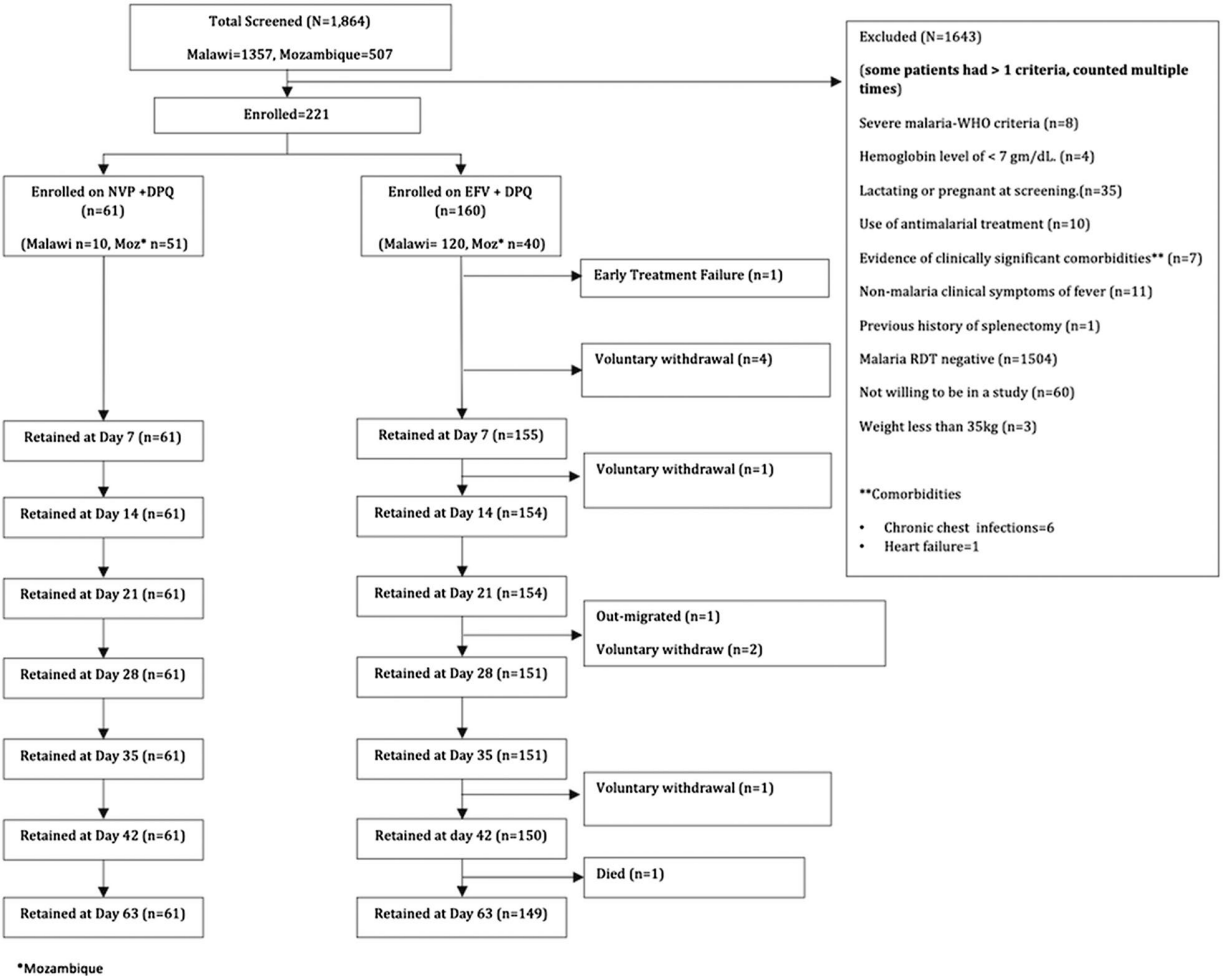
Trial profile and flow chart of participants. Trial profile and flow chart of participants enrolled into the efavirenz (EFV) and nevirapine (NVP) antiretroviral groups in Malawi and Mozambique and receiving dihydroartemisinin–piperaquine (DPQ)

A total of 1864 patients presenting at the health facilities with symptoms suggestive of malaria were screened for trial eligibility (Fig. 1). Two hundred and twenty-one patients with positive *P. falciparum* malaria blood met the eligibility criteria and were enrolled in the trial. None of the participants on nevirapine-ART and 5.6% (9/160) on efavirenz-ART were lost-to-follow-up or withdrew consent.

### Baseline characteristics

The baseline characteristics of enrolled study participants are summarized in Table 1. Most of the participants in the nevirapine-ART group (83.6%, N = 61) were from Mozambique while those on efavirenz-ART were predominantly from Malawi (75%, N = 160). Participants in the nevirapine-ART group were older and had been on ART for a longer period than those in the efavirenz-ART group. The geometric mean parasite density and median CD4 cell count were higher in the nevirapine-ART group than in the efavirenz-ART arm. A lower proportion of participants on nevirapine-ART were on cotrimoxazole prophylaxis compared with those on efavirenz-ART. There were no major differences between the two groups in body mass index and hemoglobin concentrations. The median Fridericia-corrected QT (QTcF) interval at recruitment was longer in the nevirapine-ART group than the efavirenz-ART group.

**Table 1:** Baseline characteristics of enrolled participants in Malawi and Mozambique, stratified by group of antiretroviral therapy

Variable	Efavirenz group (N = 160)	Nevirapine group (N = 61)
Age in years, median (IQR)	38.6 (32.3–44.7)	47.0 (37.9–51.8)
Female, %	107 (66.9)	46 (75.4)
Body mass index, kg/m^2^, median (IQR)	20.8 (19.3–22.7)	21.1 (18.5–23.9)
WHO BMI classification, n (%)		
Underweight	26 (16.3)	15 (24.6)
Normal	123 (76.8)	35 (57.4)
Overweight	5 (3.1)	6 (9.8)
Obese	6 (3.8)	5 (8.2)
Duration on ART in months, median (IQR)	11 (5–32)	55 (33–70)
Presenting symptoms, n (%)		
Fever	146 (91.3)	57 (93.4)
Headache	144 (90.0)	57 (93.4)
Body aches	89 (55.6)	41 (67.2)
Nausea	40 (25.0)	33 (54.1)
Axillary temperature at enrolment, median (IQR)	37.7 (37.0–37.9)	37.1 (36.6–37.8)
Geometric Mean Parasite density, 95% CI.	2681 (1964–3661)	9819 (6606–14,593)
Parasite density > 2000 parasites/µL, n (%)	84 (52.5)	53 (86.9)
Pre-treatment hemoglobin concentration, g/dL, median (IQR)	12.2 (10.9–13.5)	11.9 (10.7–12.7)
Pre-treatment CD4 cell count, median (IQR) cells/mm^3^	256 (140–360)	390 (237–500)
CD4 cell count < 350, n (%)	106 (66.3)	27 (44.3)
Median (IQR) QTcF interval (ms)	395 (360–418)	418 (389–438)
Reported use of any insecticide-treated bed net before presentation, % (n)	44 (27.5)	16 (26.2)
Current use of cotrimoxazole prophylaxis, % (n)	109 (68.1)	12 (19.6)

### Treatment dosage and tolerability

The total median (range) dosages of dihydroartemisinin and piperaquine administered to participants were 7.0 mg/kg (5.1–9.9) and 56.3 mg/kg (40.9–79.1), respectively in the efavirenz-ART group and 7.0 mg/kg (5.6–8.6) and 55.7 mg/kg (45.0–68.6), respectively in the nevirapine-ART group. The dosages were well tolerated: 3 participants in the efavirenz-ART group and none in the nevirapine-ART group vomited following intake of DPQ. Participants who vomited were re-dosed and none was withdrawn from the study due to persistent vomiting.

### Treatment efficacy

As shown in Table 2, only one participant in the efavirenz-ART group, with baseline CD4 count of 26 cells/μL, had ETF on treatment day 2. Also, seven cases in the efavirenz-ART group had LPF. Parasite genotyping results were available in 6 of the 7 LPF cases which were all classified as re-infections. No treatment failures occurred in the nevirapine-ART group.

**Table 2:** Efficacy outcomes and adequate clinical and parasitilogical response rates by day 42 among the enrolled participants stratified by group of antiretroviral therapy

Variable	DPQ + efavirenz N = 160	DPQ + nevirapine N = 61
Early treatment failure-no. (%)	1 (0.6)	0
Development of danger signs or severe malaria	0	0
Parasitaemia on day 2 greater than day 0	0	0
Parasitemia on day 3 ≥ 25% of count on day 0	1 (0.6)	0
Parasitemia on day 3 with axillary temperature ≥ 37.5 °C	0	0
Late clinical failure-no. (%)	0	0
Late parasitological failure-no. (%)	7 (4.4)	0
Recrudescence	0	0
Reinfection	6 (3.8)	0
Indeterminate or sample unavailable	1 (0.6)	0
Adequate clinical and parasitolocal response rates by different scenarios		
Intention-to-treat analysis		
PCR-corrected cure rate-% (95% CI)		
Scenario 1^a^	99.4 (95.6–99.9)	100
Scenario 2^b^	93.1 (88.0–96.2)	100
PCR-uncorrected cure rate-% (95% CI)		
Scenario 1^a^	95.6 (90.3–97.5)	100
Scenario 2^b^	89.4 (83.5–93.3)	100
Scenario 3^c^	89.9 (82.3–94.4)	100
Scenario 4^d^	89.8 (77.4–95.8)	100
Per-protocol analysis		
Number of patients	151	61
PCR-corrected cure rate-% (95% CI)		
Scenario 5^e^	99.3 (95.4–99.9)	100
Scenario 6^f^	98.8 (95.1–99.7)	100
PCR-uncorrected cure rate-% (95% CI)		
Scenario 5^e^	95.3 (90.5–97.8)	100
Scenario 6^f^	94.7 (89.7–97.3)	100

^a^Scenario 1: Indeterminate or unavailable PCR samples or loss to follow up by day 42 (n = 9, in efavirenz-ART group) in intention-to-treat population treated as treatment success

^b^Scenario 2: Indeterminate or unavailable PCR samples or loss to follow up by day 42 (n = 9, in efavirenz-ART group) in the intention-to-treat population treated as treatment failures

^c^Scenario 3: On cotrimoxazole prophylaxis in the intention-to treat analysis and combined with scenario 2 above

^d^Scenario 4: Not on cotrimoxazole prophylaxis in the intention-to treat and combined with scenario 2 above

^e^Scenario 5: Indeterminate or unavailable PCR samples in per-protocol population treated as treatment success

^f^Scenario 6: Indeterminate or unavailable PCR samples in per-protocol population treated as treatment failure

In the ITT analyses, the PCR-corrected day 42 ACPR was 99.4% (95% confidence interval [CI] 95.6–99.9%) in the efavirenz-ART group and 100% in the nevirapine-ART group (see Additional file 1). The day 42 PCR-uncorrected ACPR was 95.6% (95% CI 90.3–97.5%) in the efavirenz-ART group and 100% in the nevirapine-ART group (see Additional file 2).

In the PP analyses, the day 42 PCR-corrected ACPR was 99.3% (95% CI 95.4–99.9%) in the efavirenz-ART group and 100% in the nevirapine-ART group. The day 42 PCR-uncorrected ACPR was 94.7% (95% CI 89.7–97.3%) in the efavirenz-ART group and 100% in the nevirapine-ART group.

In sensitivity analyses which considered missed visits or samples, unavailable PCR results and loss to follow up as treatment failures, the day-42 PCR-corrected ACPR was 93.1% (95% CI 88.0–96.2%) in the ITT population and 98.8% (95% CI 95.1–99.7%) in the PP population. Details of the different scenarios accounting for the missing results are shown in Table 2.

### Parasite clearance time

**Fig. 2:**
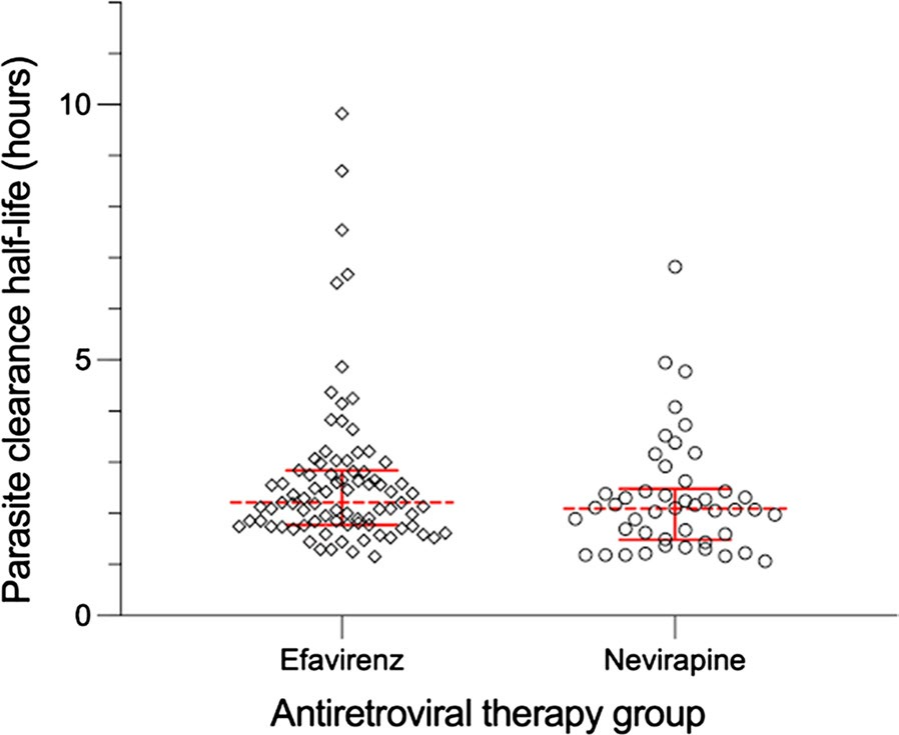
Parasite clearance half-life. Parasite clearance half-life in the intention-to-treat population stratified by antiretroviral therapy group [efavirenz (diamond) and nevirapine (circle)]. Red dotted middle line is the median parasite clearance rate, lower and upper red solid bars represent the interquartile range

Parasite clearance parameters were calculated in 46 and 84 participants in the nevirapine-and efavirenz-ART groups, respectively, who had detectable parasitemia at two or more post-dosing time points. The median (range) PC_50_ and the median (range) PC_90_ in the ITT population were 3.1 (0.2–10.3) h and 8.2 (2.4–19.7) h, respectively, in the nevirapine-group and 4.2 (0.6–40.3) h and 10.1 (3.2–63.1) h, respectively, in the efavirenz-group. The median parasite clearance half-life (range) were 2.1 (1.1–6.8) and 2.2 (1.2–9.8) h in the nevirapine- and efavirenz-ART groups, respectively (Fig. 2). One participant (2.2% [95% CI 0.3–14.8]) in the nevirapine-ART group and five participants (6.0% [2.5–13.7]) in the efavirenz-ART group, had parasite clearance half-life of > 5.5 h. All six participants had a baseline parasite density of > 3500 parasites/µL and CD4 cell count of < 250 cells/mm^3^. None of these six participants experienced malaria recurrence during the follow up period.

### Day-7 plasma piperaquine concentrations

On day 7 of follow-up, blood samples for plasma piperaquine quantification were available from 179 of the retained 216 participants. Of these, 62.0% (111/179) had day-7 piperaquine concentrations that were below the LLOQ (< 25 ng/mL): 60.0% (81/135) in the efavirenz-ART group and 68.2% (30/44) in the nevirapine-ART group. The piperaquine values for these individuals were imputed to half the lower limit of quantification. However, since these values were more than > 10% of the data, they were excluded in calculation of median piperaquine exposure. Overall, the median (range) day-7 piperaquine concentration in participants with concentrations > LLOQ was 57 (25.5–592.8) ng/mL in the efavirenz-ART group and 79.1 (25.4–211.1) ng/mL in the nevirapine-ART group. After excluding < LLOQ values, participants who experienced malaria recurrence by day 42 (n = 3) had a median (range) day-7 piperaquine concentration of 63.9 (49.7–78.9) ng/mL and this value was 66.7 (25.4–592.8) ng/mL in participants who did not experience malaria recurrence (n = 65, p = 0.965).

### Fever clearance

At baseline, 58.1% (93/160) of participants in the efavirenz-ART group and 31.1%, (19/61) in the nevirapine-ART group were febrile (axillary temperature ≥ 37.5 °C). The median fever clearance time (IQR) was 6 h (6–60) in the efavirenz-ART group and 6 h (6–12) in the nevirapine-ART group. By treatment day 2, only 3.8% (6/160) participants in the efavirenz-ART group and 1.6% (1/61) in the nevirapine-ART group were febrile.

### Safety

From enrolment to follow-up day 63, there were 69 adverse events of special interest (AESIs) (54 and 15 in the efavirenz-ART group and nevirapine-ART group, respectively) and 12 serious adverse events (SAEs) (10 and 2 in the efavirenz-ART group and nevirapine-ART group, respectively) (Table 3). None of the SAEs and AESIs were judged to be definitely related to DPQ. Forty-eight (30.0%) of the AESIs in the efavirenz-ART group and 8 (13.1%) in the nevirapine-ART group were judged to be probably related to DPQ (Table 3). These were mostly cases of prolonged QTcF interval. One death occurred on day 59 in a participant on efavirenz-ART who had been treated for deep vein thrombosis from day 33 and had CD4 count persistently below 50 since enrolment. Additional file 3 provides details on the SAEs that occurred during follow up. Except for the death, all participants with AESIs and SAEs recovered without sequelae.

**Table 3:** Summary of serious adverse events and adverse events of special interest stratified by antiretroviral group

Type of adverse event	DPQ and efavirenz-based ART regimen N = 160	DPQ and nevirapine-based ART regimen N = 61
	n^a^ (%) [#]^b^	n^a^ (%) [#]^b^
Serious adverse events (SAEs)	8 (5.0), [10]	2 (3.3), [2]
Adverse event(s) of special interest (AESI)	54 (33.8), [54]	15 (24.6), [15]
Fridericia corrected QT prolongation^c^	48 (30.0)	8 (13.1)
Palpitations	4 (2.5)	2 (3.3)
Dizziness	2 (1.3)	2 (3.3)
Urticaria	0	1 (1.6)
Itchiness	0	1 (1.6)
Chest pain	0	1 (1.6)
Drug-related adverse events (SAEs)		
Not related	7 (4.4), [9]	2 (3.3), [2]
Possibly related	1 (0.6), [1]	0
Probably related	0	0
Definitely related	0	0
Drug-related adverse events (AESI)		
Not related	1 (0.6), [1]	4 (6.6) [4]
Possibly related	5 (3.1), [5]	3 (4.9), [3]
Probably related	48 (30.0), [48]	8 (13.1), [8]
Definitely related	0	0

^a^n The number of participants that experienced the event; % is percentage of participants experiencing that event in the intention-to-treat population

^b^# The total number of events that occurred

^c^Fridericia corrected QT prolongation defined as change in QTc interval of > 60 ms from baseline to day 2 (last day) of antimalarial treatment, also reported separately (in detail) in Table 4

### Haematological parameters

The mean (SD) haemoglobin concentration decreased from baseline to day 7 (efavirenz-ART group: 12.2 to 11.7 g/dL, p < 0.001 and nevirapine-ART group: 11.7 to 10.7 g/dL, p < 0.001), but increased thereafter up to day 42, from 11.7 to 12.4 g/dL in the efavirenz-ART group (p < 0.001) and from 10.7 to 11.6 g/dL in the nevirapine-ART group (p < 0.001). Following DPQ treatment, the median (IQR) CD4 cell count increased from baseline to day 28, from 257 (140–357) to 320 (216–521) in the efavirenz-ART group (p < 0.001) and from 390 (204–500) to 429 (204–580) in the nevirapine-ART group (p = 0.133).

### QT interval abnormalities

Data on baseline QTcF interval were available for 218 participants; 158 and 60 in the efavirenz-ART and nevirapine-ART group, respectively. The proportions of participants with predose QTcF ≥ 450 ms were 3.8% (6/158) in the efavirenz-ART group and 11.7% (7/60) in the nevirapine-ART group. Only 4 participants in the efavirenz-ART group had missing QTcF values on day 2 (last day of treatment). A change in QTcF interval of > 60 ms from baseline to day 2 occurred in 31.2% (48/154) and 13.3% (8/60) in the efavirenz and nevirapine groups, respectively (Table 4). No participant had an absolute day 2 QTcF interval ≥ 500 ms. Observed QTcF interval abnormalities resolved by day 7 or 14 of follow up. No cardiovascular abnormalities were detected in these individuals.

**Table 4:** Median Fridericia corrected QT interval (QTcF), and proportion with abnormal ECG findings from baseline to last day of dosing among participants in the efavirenz- (N = 158) and nevirapine- (N = 60) based antiretroviral therapy groups

Time of ECG test	Median (IQR) QTcF in ms		Proportion with QTcF ≥ 450 ms		Proportion with QTcF change > 60 ms from baseline to last day of dosing on day 2 (taken within 2 h of dosing)^a^	
	Efavirenz-based ART group	Nevirapine-based ART group	Efavirenz-based ART group	Nevirapine-based ART group	Efavirenz^b^-based ART group	Nevirapine^b^-based ART group
	NA	NA	n (%)	n (%)	n (%)	n (%)
Day 0	395 (360–418)	418 (390–438)	6 (3.8)	7 (11.8)	NA	NA
Day 1	403 (383–423)	418 (388–437)	10 (6.3)	6 (10.0)	19 (12.3)	1 (1.7)
Day 2	424 (408–442)	434 (414–459)	29 (18.4)	21 (35.0)	48 (31.2)	8 (13.3)
Follow up visit after day 2 (day 7 or 14 of follow up)	410 (384–436)	424 (402–437)	0	0	NA	NA

*IQR* interquartile range, *ECG* electrocardiogram, *ART* antiretroviral drug, *NA* not applicable

^a^Adverse event of special interest

^b^N = 154 in Efavirenz group and 60 in the Nevirapine group

## Discussion

The WHO recommends the use of first-line anti-malarial drugs with PCR-corrected ACPR of > 90%. In this study, the day 42 PCR-corrected ACPR for DPQ exceeded 99% among adult HIV-infected patients with uncomplicated malaria concurrently taking efavirenz- or nevirapine-based ART; the lower limit of the 95% CI for the DPQ ACPR exceeded 95%. Rapid parasite clearance, similar to that in HIV-uninfected individuals [28–31] was achieved, irrespective of the type of ART taken by the study participants. Malaria reinfections occurred in the efavirenz-ART group but, overall, day 42-PCR uncorrected ACPR remained high in both ART groups, suggesting that DPQ had a good prophylactic effect against malaria reinfections. DPQ treatment also resulted in rapid resolution of fever and marked improvement in haemoglobin concentrations irrespective of ART as well as improvement in CD4 cell count, especially in those taking efavirenz-based ART.

An anti-malarial treatment response depends on host immunity and anti-malarial drug blood concentrations. Efavirenz has been associated with induction of CYP3A4 and CYP2B6 enzymes while nevirapine has been associated with inhibition or sometimes induction of the enzymes [5]. Previous pharmacokinetic studies found that, compared with antiretroviral naïve HIV-infected individuals, those taking DPQ plus efavirenz-ART had lower piperaquine area under the concentration–time curve (AUC) [8, 32] while those taking DPQ plus nevirapine-ART had higher piperaquine AUC [8]. Similarly, other studies found that when co-administered with efavirenz or nevirapine, ACT resulted in altered pharmacokinetics of artemisinin and its metabolites [33–37]. The finding of high efficacy of DPQ suggests that any alterations in the pharmacokinetics of dihydroartemisinin or piperaquine due to efavirenz or nevirapine had limited clinical significance in this study population. However, this needs further confirmation in future studies which would correlate PK data with observed efficacy endpoints.

To date, no previous study has assessed the efficacy and safety of DPQ in malaria-HIV coinfected non-pregnant adults on ART. A previous Tanzanian study [38] found that HIV-infected adults with uncomplicated malaria on efavirenz-ART who were treated with artemether–lumefantrine had a lower PCR-uncorrected day 28 ACPR (82.5%) than HIV-infected antiretroviral naïve individuals (94.5%), while those on nevirapine-based ART had a higher day 28 PCR-uncorrected ACPR (97.6%). No cases of ETF were detected in a study population in which almost half of the participants had CD4 cell counts < 350. Thus, the study showed high efficacy of artemether and its metabolites (responsible for the initial clearance of malaria parasite [39, 40]) despite ART co-administration, which is consistent with our study findings. However, in contrast to our findings, the Tanzanian study found high rates of recurrent malaria in those on efavirenz-ART. This is likely to reflect the longer half life and hence superior prophylactic effect of the ACT-partner drug, piperaquine, in this study compared with lumefantrine in the previous study.

Day-7 plasma ACT concentrations have been proposed as a marker of overall exposure of the longer acting partner drug of ACT and have been shown to be predictive of treatment efficacy by day 28 after malaria treatment [22]. In the present malaria-HIV co-infected sub-population, most participants had day-7 piperaquine concentrations that were below the LLOQ. However, in participants who had piperaquine concentration values > LLOQ, the piperaquine concentrations were not significantly different between those who experienced malaria recurrence by day 42 and those who did not. The observed < LLOQ concentrations could be due to increased metabolism of piperaquine, as a result of efavirenz induction of CYP3A4 enzymes, or a limitation in the HPLC assay to detect smaller concentrations of piperaquine. Nevertheless, the observed high efficacy of DPQ highlights that any PK interaction between efavirenz or nevirapine and piperaquine did not predict the clinical outcomes in this study.

Delayed parasite clearance (parasite clearance half-life of > 5.5 h) has been shown to be associated with resistance of the parasites to artemisinins [41]. Although a smaller proportion of participants in both ART groups had parasite clearance half-life of > 5.5 h, they did not experience malaria recurrence in the follow up period, despite having most participants with piperaquine concentrations below the lower limit of quantification. Notably, they all had a low baseline CD4 cell count with a relatively higher malaria parasite load compared to the rest of the participants. This higher parasite load coupled with low immunity at presentation could explain the delayed clearance of malaria parasites that was observed in these participants.

Treatment-emergent AESIs occurred in nearly a third and one-quarter of the participants on efavirenz- and nevirapine-based ART, respectively. However, only a few cases of AESIs in the efavirenz-group and none in the nevirapine-group were judged to be possibly associated with DPQ (Table 3). QTcF prolongation of at least 60 ms from baseline to the last treatment day did occur in a sizable proportion of participants but none had an absolute QTcF interval of 500 ms and there were no clinically detectable events. The QTcF prolongation resolved spontaneously by day 14. The observed QTcF prolongation may have been due to fever resolution [42, 43] rather than piperaquine, but whatever the mechanism, we can conclude that DPQ can be safely used in this group of patients. This observation is also in line with WHO’s recommendation that there is no evidence of increased risk of cardiotoxicity following exposure to current doses of DPQ for treatment of uncomplicated malaria [44]. Additionally, a similar phenomenon, of prolonged QTc interval which resolves following recovery from malaria, has been previously observed in 17.1% (n = 152) of adults living with HIV and on efavirenz-based ART who were treated for uncomplicated malaria with artemether–lumefantrine in Zambia [10]. Nevertheless, this needs to be confirmed in future studies of DPQ use in malaria-HIV coinfected adults on efavirenz- or nevirapine based ART.

The major strengths of this study were the directly-supervised DPQ dosing, large sample size of participants on efavirenz-based ART and minimal loss to follow-up (< 5%). However, the required sample size was not achieved in the nevirapine-based ART group because the national ART programme phased out this regimen during the course of the study. Nevertheless, participants in this group had high baseline parasite densities which permitted accurate assessment of DPQ’s parasite clearance and prophylactic efficacy.

Nearly one-half of enrolled study participants in the efavirenz group had parasite densities of < 2000/mm^3^ and nearly two-thirds were on cotrimoxazole prophylaxis which has some antimalarial effects [45, 46]. Thus, it can be argued that the high ACPR found in the efavirenz-group could have partly been due to the anti-malarial effects of cotrimoxazole and immunity-mediated clearance of low density parasitaemias. However, day-42 PCR-corrected and uncorrected ACPR did not significantly vary according to the use of cotrimoxazole prophylaxis or baseline parasite density. In addition, at least two-thirds of participants in this group had CD4 cell count < 350/mm^3^ which is likely to have compromised their ability to clear parasites and prevent reinfections [2, 3, 25]. In spite of this, DPQ achieved high ACPR in this group suggesting its high therapeutic and prophylactic effectiveness.

In this study, the day-42 PCR corrected and uncorrected ACPR appeared to be higher among individuals on nevirapine-based ART than among those on efavirenz-based ART. However, this study was not designed to compare the efficacy and safety of DPQ between the two groups and the full sample size was not achieved in the nevirapine arm. It is therefore inappropriate to make direct comparison of DPQ efficacy between individuals taking the two ART regimens.

## Conclusion

This study found that dihydroartemisinin–piperaquine was highly efficacious and safe when used to treat uncomplicated *P. falciparum* malaria in individuals taking efavirenz or nevirapine-based ART. A higher than expected observed cases of QTc interval prolongation (> 60 ms from baseline to day 2) following treatment with dihydroartemisinin–piperaquine were observed and thought to likely be due to resolution of fever as part of the malaria recovery process. Under the “HIV Test and Treat” approach, many HIV-infected individuals in high burden countries will initiate ART early before they are severely immunosuppressed and this study supports the use of dihydroartemisinin–piperaquine for the treatment of uncomplicated malaria in such patients.

## Additional files

**Additional file 1.** Day 42 PCR-corrected efficacy plot. Kaplan–Meier survival plot of participants who were treated with dihydroartemisinin–piperaquine (DHA PPQ) in the efavirenz (EFV)- and nevirapine (NVP) based antiretroviral therapy (ART) groups according to polymerase chain reaction (PCR) corrected adequate clinical and parasitological response (ACPR) by day 42 in the intention-to-treat population with loss to follow up and indeterminate or unavailable PCR samples treated as treatment success.

**Additional file 2.** Day 42 PCR-uncorrected efficacy plot. Kaplan–Meier survival plot of participants who were treated with dihydroartemisinin–piperaquine (DHA PPQ) in the efavirenz (EFV)- and nevirapine (NVP) based antiretroviral therapy (ART) groups according to polymerase chain reaction (PCR) uncorrected adequate clinical and parasitological response (ACPR) by day 42 in the intention-to-treat population with loss to follow up and indeterminate or unavailable PCR samples treated as treatment success.

**Additional file 3.** Details of serious adverse events that occurred during follow up.

## Data Availability

Data from this trial are held at the Malawi Liverpool Wellcome Trust Clinical Research Programme (MLW) which encourages optimal use of data by employing controlled access approach to data sharing with a robust system to review requests for data use and provide secure data access that is consistent with relevant ethics committee approvals. The datasets are therefore not publicly available but are available on reasonable request from the corresponding author and requests can also be initiated by contacting MLW: data@mlw.mw.

## Abbreviations

ACT: artemisinin-based combination therapy
ACPR: adequate clinical and parasitological response
ART: antiretroviral therapy
DPQ: dihydroartemisinin–piperaquine
ETF: Early treatment failure
LCF: Late clinical failure
LPF: Late parasitological failure
