# Supplementary figures and images for: Efficacy and safety of dihydroartemisinin–piperaquine for treatment of Plasmodium falciparum uncomplicated malaria in adult patients on antiretroviral therapy in Malawi and Mozambique: an open label non-randomized interventional trial
Source: Malar J. 2019 Aug 20;18:277. doi: 10.1186/s12936-019-2909-5 (PMC6700797; doi:10.1186/s12936-019-2909-5)

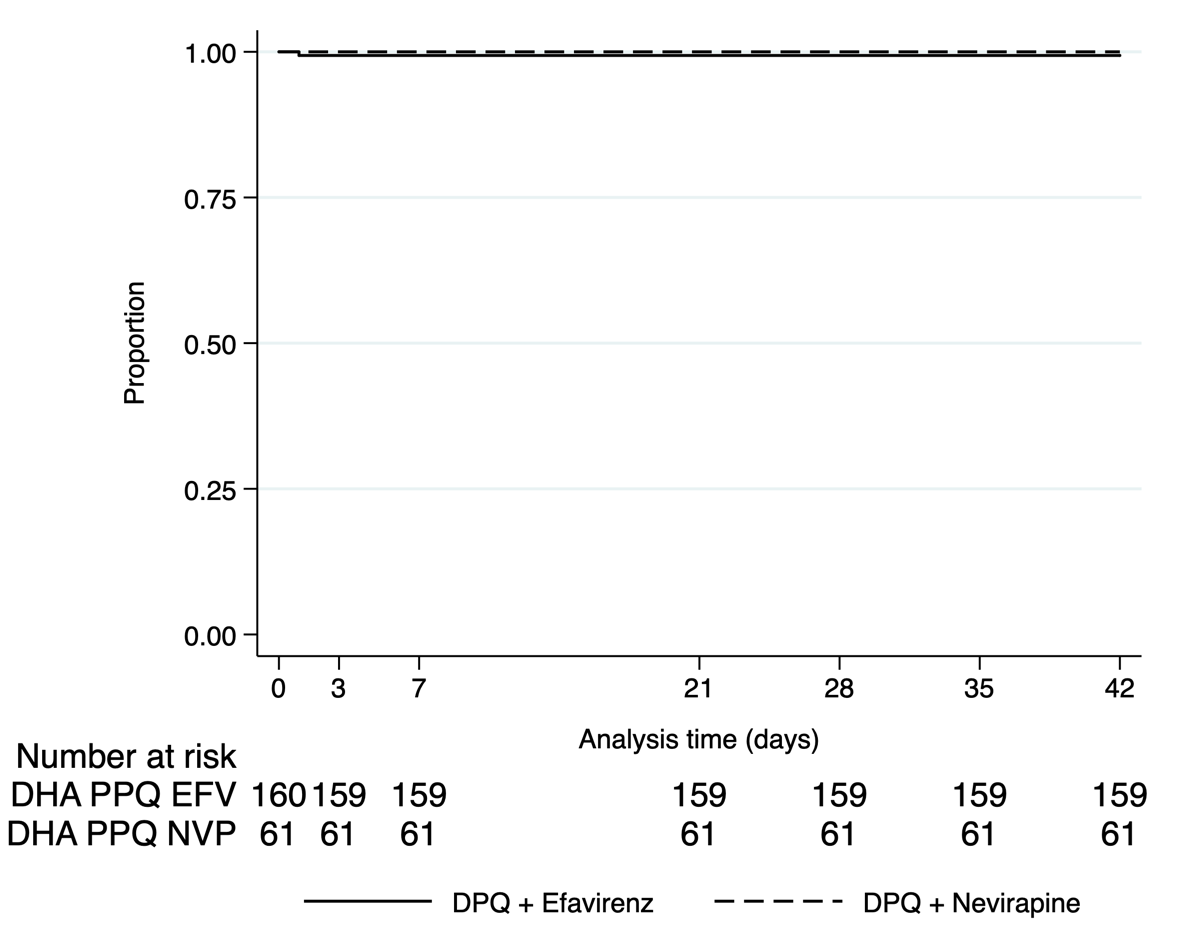

Supplement: Supplementary file 1 — Additional file 1. Day 42 PCR-corrected efficacy plot. Kaplan–Meier survival plot of participants who were treated with dihydroartemisinin–piperaquine (DHA PPQ) in the efavirenz (EFV)- and nevirapine (NVP) based antiretroviral therapy (ART) groups according to polymerase chain reaction (PCR) corrected adequate clinical and parasitological response (ACPR) by day 42 in the intention-to-treat population with loss to follow up and indeterminate or unavailable PCR samples treated as treatment success. [file 12936_2019_2909_MOESM1_ESM.png]

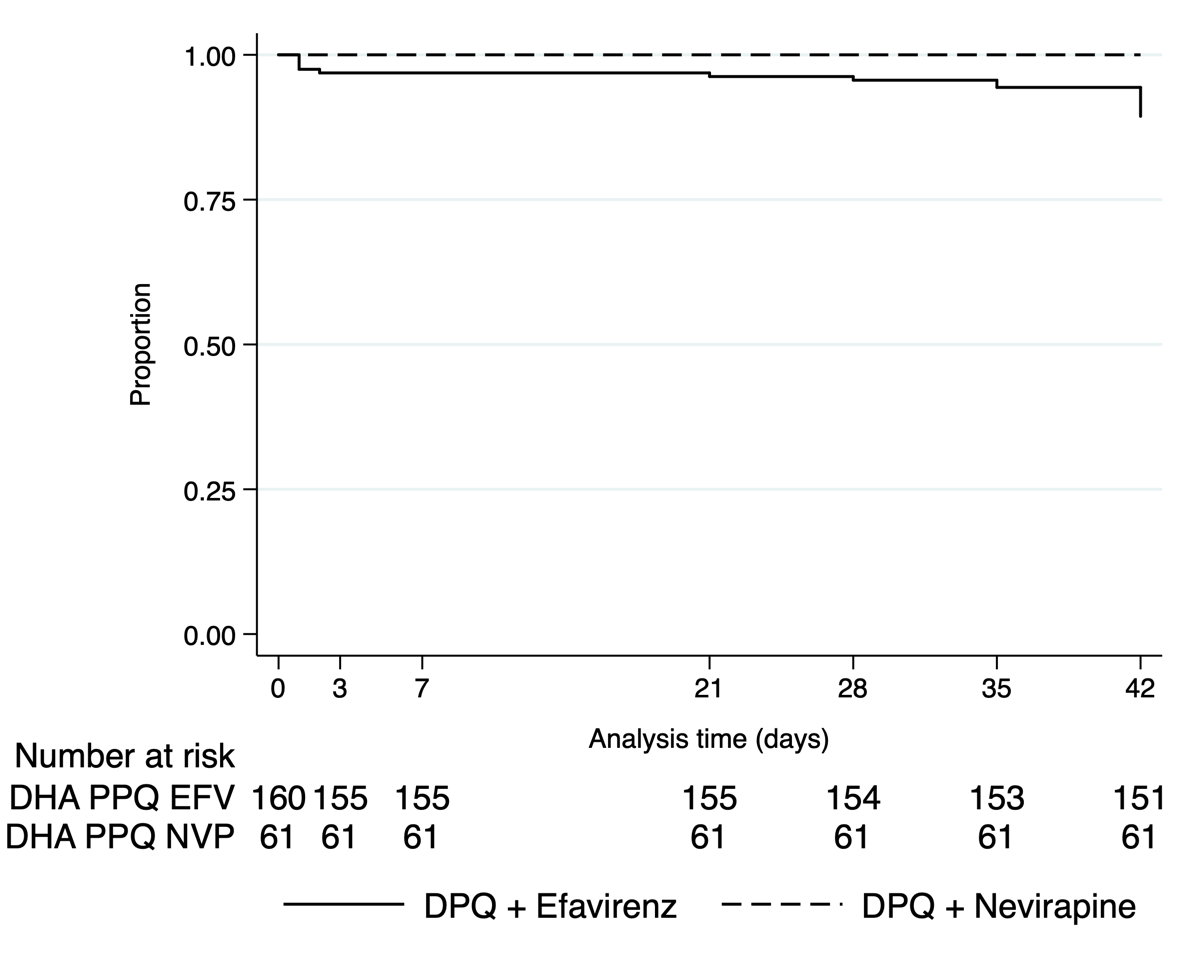

Supplement: Supplementary file 2 — Additional file 2. Day 42 PCR-uncorrected efficacy plot. Kaplan–Meier survival plot of participants who were treated with dihydroartemisinin–piperaquine (DHA PPQ) in the efavirenz (EFV)- and nevirapine (NVP) based antiretroviral therapy (ART) groups according to polymerase chain reaction (PCR) uncorrected adequate clinical and parasitological response (ACPR) by day 42 in the intention-to-treat population with loss to follow up and indeterminate or unavailable PCR samples treated as treatment success. [file 12936_2019_2909_MOESM2_ESM.png]
