# Supplementary material for: Efficacy and safety of dihydroartemisinin–piperaquine for treatment of Plasmodium falciparum uncomplicated malaria in adult patients on antiretroviral therapy in Malawi and Mozambique: an open label non-randomized interventional trial
Source: Malar J. 2019 Aug 20;18:277. doi: 10.1186/s12936-019-2909-5 (PMC6700797; doi:10.1186/s12936-019-2909-5)
Supplement: Supplementary file 3 — Additional file 3. Details of serious adverse events that occurred during follow up. [file 12936_2019_2909_MOESM3_ESM.docx]

| **Additional file 3: Details of serious adverse events experienced by participants during follow up** | | | | |
| --- | --- | --- | --- | --- |
| **Study group** | **Details of serious adverse event** | **Severity** | **Relationship to study drug** | **Resolution/outcome** |
| **Efavirenz-based antiretroviral therapy group** | Day 21 ischaemic heart disease secondary to hypertrophic obstructive cardiomyopathy. Also, evidence of chronic renal failure, anaemia and permanent left bundle branch block (dilated left atrium and ventricle). | Grade 3, required hospitalization | Possibly related to DHA-PQ. Possibly due to concomitant drugs (Efavirenz) | Resolved with sequelae, Treated with atenolol |
|  | Day 45 complicated malaria (parasite density 11,040 parasites/ microlitre) | Grade 3, required hospitalization | Unrelated | Resolved, treated with intravenous artesunate and then oral therapy artemether-lumefantrine |
|  | Day 14 neck abscess | Grade 3, required hospitalization | Unrelated | Recovered |
|  | *Day 3 acute kidney injury | G4, Life-Threatening, other medically important condition. This was also the case of early treatment which was treated as severe malaria | Unlikely due to DHA-PQ. Possibly related to concomitant drugs (Tenofavir induced kidney injury. Drug (Tenofavir was withheld and substituted with abacavir) | Resolved by day 14 |
|  | *Day 3 Grade 4 anaemia and Day 3 acute kidney injury (swollen echogenic kidney, nephritis pattern, no uraemia). Initial parasite density of 178,760 parasites/microlitre | Grade 4, Medically-important condition. | Unlikely due to study drug, possible relation to concomitant medication-Tenofavir (drug withdrawn) | Resolved by Day 14 |
|  | Day 35 severe anaemia with late parasitological failure treated with artemether-lumefantrine | Grade 4, life-threatening, medically important condition | Unlikely to DHA-PQ, unlikely to be due to concomitant drugs | Resolved by Day 63 |
|  | Day 3 acute kidney injury, with no uraemia. Had severe anaemia and high baseline parasite density (123360 parasites/microlitre) which was cleared by day 3 | Grade 4, life-threatening. | Unrelated to DHA-PQ | Resolved by day 14 |
|  | ¶ Day 33 ultrasound scan (USS) confirmed deep vein thrombosis (DVT), had a low baseline CD4 count, previously treated for cellulitis | Grade 2, medically-important condition requiring hospitalization | Unrelated to DHA-PQ, unrelated to concomitant medication | Resolved DVT, treated with sub-cutaneous heparin, hospitalized for 10 days and discharged on Day 43 |
|  | ¶ Day 59 Death in participant who presented on day 33 above with USS confirmed DVT | Fatal | Unlikely to DHA-PQ, unlikely to concomitant drugs (doses of medications were not changed) | Fatal |
|  | Severe pneumonia, previous history of pulmonary tuberculosis. Had grade 2 thrombocytopenia, low blood pressure. CD4 count of 116 and grade 4 transaminases. Final working diagnosis of pulmonary tuberculosis (PTB) relapse | Grade 3, prolongation of existing hospitalization | Unrelated to DHA-PQ, unrelated to concomitant medication | Recovered following PTB re-treatment |
| **Nevirapine-based antiretroviral therapy group** | Day 30, dislocated shoulder | Severe, no hospitalisation required, treated as outpatient | Unlikely | Recovered by Day 63 |
|  | Day 35 Pulmonary TB relapse, anaemia and thrombocytopenia | Life threatening | Unlikely | Was still on treatment by Day 63, was followed up until completion of treatment. Participant recovered |
| * Events occurred in same participant | |  |  |  |
| ¶ Events occurred in same participant  DHA=PQ: dihydroartemisinin-piperaquine | |  |  |  |
